# The Effect of Sex and Performance Level on Pacing in Duathlon

**DOI:** 10.3390/sports6040152

**Published:** 2018-11-23

**Authors:** Pantelis T. Nikolaidis, Elias Villiger, Rodrigo L. Vancini, Thomas Rosemann, Beat Knechtle

**Affiliations:** 1 Exercise Physiology Laboratory, Nikaia 18450, Greece; 2 Institute of Primary Care, University of Zurich, Zurich 8091, Switzerland; evilliger@gmail.com (E.V.); thomas.rosemann@usz.ch (T.R.); beat.knechtle@hispeed.ch (B.K.); 3 Center of Physical Education and Sport, University of Espirito Santo, Vitoria 29040-090, Brazil; rodrigoluizvancini@gmail.com; 4 Medbase St. Gallen am Vadianplatz, St. Gallen 9001, Switzerland

**Keywords:** cycling, running, sex difference, ultra-endurance

## Abstract

The purpose of the present research was to study the effect of sex and performance on pacing in short (Run1-10 km, Bike-50 km and Run2-5 km) and long distance (Run1-10 km, Bike-150 km and Run2-30 km) in the Powerman World Championship ‘Powerman Zofingen’. All finishers (*n* = 6671; women, *n* = 1037; men, *n* = 5634) competing either in the short or long distance versions of ‘Powerman Zofingen’ from 2003 to 2017 were analyzed for the time spent in each discipline (Run1, Bike and Run2), and in transition (Tran) from Run1 to Bike (Tran1) and from Bike to Run2 (Tran2). Athletes were ranked in quartile (Q) groups (Q1, Q2, Q3 and Q4), with Q1 the fastest and Q4 the slowest. In short distance, in both sexes, a medium discipline/transition × quartile interaction on relative time was observed (*p* < 0.001, η^2^_p_ = 0.103 and η^2^_p_ = 0.119, respectively), where Q1 was relatively the fastest in Tran1, Tran2 and Run2, and the slowest in Bike (*p* < 0.001). In long distance, in both sexes, a large discipline/transition × quartile interaction on relative time was observed (*p* < 0.001, η^2^_p_ = 0.208 and η^2^_p_ = 0.180, respectively), where Q1 was relatively the fastest in Tran1, Tran2 and Run2, and the slowest in Bike (*p* < 0.001). In summary, a similar trend of variation of pacing by performance level was observed in both sexes and distances with the fastest duathletes being the fastest in Run2 and both transitions, and the slowest in Bike.

## 1. Introduction

Duathlon is an endurance multi-sport which includes running (Run1), followed by cycling (Bike) and again running (Run2), interspersed by respective transitions, i.e., from Run1 to Bike (Tran1) and from Bike to Run2 (Tran2). The distance of the total race and of each discipline might vary, e.g., 5 km Run1-20 km Bike-2.5 km Run2 [1], 5-30-10 km [2] and 5-40-5 km [3].

‘Powerman Zofingen’ is a popular duathlon including two versions, a short distance (10-50-5 km) and a long distance (10-150-30 km) as a part of the International Triathlon Union (ITU) Powerman Long Distance Duathlon World Championships [4]. As an endurance sport, performance in this multi-sport depends on aerobic capacity, which has been suggested by research showing high scores of maximal oxygen uptake (VO_2_max) in duathletes, and a very large inverse correlation of race time with VO_2_max [5]. Aerobic capacity has also been shown as the best predictor of race time in duathlon [6]. In addition to the above-mentioned physiological characteristics, performance in duathlon might be related to pacing, as has been indicated by research in other multi-sports [7] and endurance sports [8].

Pacing is the strategy where effort is managed during an exercise bout considering a specific goal and the likely demands of the task [9]. It can be also defined as the process where the overall energy demand of an exercise is regulated on a moment-to-moment basis, to ensure that the exercise can be completed in a minimum time and without a catastrophic biological failure [10]. Accordingly, the speed can increase, decrease or maintain across a race; it can achieve a maximal (inverse U shape); or it can show a combination of decrease and increase (U shape), or multiple fluctuations [11].

In the case of duathlon, pacing might be evident in the distribution of effort in each discipline (i.e., Run1, Bike and Run2) as quantified by their relative (%) contribution to the total race time. The distribution of effort in disciplines (Swim, Bike1, Bike2 and Run) has been previously examined in ‘Ultraman Hawaii’ [7], where the fastest triathletes were relatively (%) faster in cycling and running than in swimming. In addition, it was shown that the fastest men were relatively faster in running than in cycling, whereas their female counterparts performed similarly in these two disciplines [7], indicating that pacing might vary by both sex and performance.

It was acknowledged that several measures of pacing were previously used by studies in endurance and ultra-endurance races [7,12,13,14]. For instance, the speed across 10 km splits—expressed as percentage of the speed in the first 10 km split—was examined in a 100 km ultra-marathon [12]. Furthermore, pace range [14] and coefficient of variation of speed [13] have been used to describe pacing in marathon races. For the purpose of the present study, pacing was considered as the relative contribution (%) of each discipline and transition (i.e., the time needed once finished a discipline until the start of the next one) of duathlon. This definition of pacing was in agreement with the above-mentioned definition of Baron et al. [10]. Particularly, the focus of the present study was on the regulation of the energy demand of disciplines and transitions, that is, whether duathletes would perform differently in disciplines/transitions depending on their sex and performance.

To the best of our knowledge, no previous study has ever examined the effect of performance level on pacing in duathlon. Considering the increasing popularity of this multi-sport [15], information about the variation of pacing strategies by performance level would be of practical value for coaches and fitness trainers working with duathletes. In addition, as ‘Powerman Zofingen’ has two versions differing by distance, it offers a model to investigate whether a potential effect of performance on pacing varies by distance. Based on findings from triathlon, it was hypothesized that sex and performance level would also influence pacing in duathlon [7]. Therefore, the purpose of the present research was to study the effect of performance level on pacing in the short and long distance ‘Powerman Zofingen’. According to the adopted definition of pacing, the outcome measures were relative times (%), where a lower score in a discipline/transition denoted a relatively faster performance and vice versa, independently from the corresponding absolute scores.

## 2. Materials and Methods

### 2.1. Study Design and Participants

All procedures of the study were approved by the Institutional Review Board of Kanton St. Gallen, Switzerland. Since the study involved the analysis of publicly available data (1 June 2010), there was a waiver of the requirement for informed consent of the participants. The study was conducted in accordance with recognised ethical standards derived from the Declaration of Helsinki as revised in 2013.

Data were derived from the ‘Powerman Zofingen’ and the ‘ITU Powerman Long Distance Duathlon World Championships’. We examined the effect of sex and performance on pacing (i.e., the relative contribution—%—of each discipline and transition times—Transition 1 and Transition 2—to overall race time) of duathletes competing either in the short (i.e., 10-50-5 km) or the long distance (i.e., 10-150-30 km) race. A duathlon includes three parts; running, cycling and running, which are carried out in sequence.

Since 2002, the long distance race of ‘Powerman Zofingen’ has had the sequence of 10 km running, 150 km cycling and 30 km running, whereas the short distance includes 10 km running, 50 km cycling and 5 km running. Both races take place at the same time. In short distance, 556 women (age 34.7 ± 9.0 years) and 2945 men (39.6 ± 10.5 years) were analysed, whereas in long distance, 481 women (38.7 ± 8.7 years) and 2689 men (40.4 ± 9.5 years) were considered.

### 2.2. Procedures

Data were obtained from the official website of ‘Powerman Zofingen’ www.powerman.ch/de. Considering their overall race time (min), athletes were ranked in four quartile groups (Q1, Q2, Q3 and Q4) with Q1 the fastest and Q4 the slowest within each sex. In the short distance, cut-offs for performance quartiles were Q1 ≤ 169.7 min, 169.7 < Q2 ≤ 183.4 min, 183.4 < Q3 ≤ 199.8 min and Q4 > 199.8 min in women, and Q1 ≤ 157.3 min, 157.3 < Q2 ≤ 168.7 min, 168.7 < Q3 ≤ 183.7 min and Q4 > 183.7 min in men. In the long distance, cut-offs for performance quartiles were Q1 ≤ 498.8 min, 498.8 < Q2 ≤ 541.2 min, 541.2 < Q3 ≤ 584.1 min and Q4 > 584.1 min in women, and Q1 ≤ 461.1 min, 461.1 < Q2 ≤ 499.6 min, 499.6 < Q3 ≤ 542.8 min and Q4 > 542.8 min in men. The classification into quartile groups was based upon an aggregate of all data instead of a year-by-year analysis, since it has been observed recently that race times in duathlon did not change from 2003 to 2017 [15]. Performance (i.e., relative time) in each discipline (i.e., Run1, Bike and Run2) and transition (i.e., Transition1 and Transition2) was expressed as percentage of the total race time using the formula ‘100 × disciplines’ time/total time’.

### 2.3. Statistical Analysis

Data are presented as mean ± standard deviation. Within each distance, a between-within measures analysis of variance (ANOVA) examined the main effects of sex, performance group and discipline/transition, and the sex × discipline/transition and performance group × discipline/transition interaction on relative time. The Bonferroni post-hoc test examined differences among performance groups. The magnitude of these main effects and interactions was examined using effect size partial eta square (η^2^_p_), and was evaluated as following: small (0.010 < η^2^_p_ ≤ 0.059), moderate (0.059 < η^2^_p_ ≤ 0.138) and large (η^2^_p_ > 0.138) [16]. The race distance was not considered as an independent variable, since the distances of disciplines were not proportional between the short and the long distance (i.e., the ratio was 1 for Run1, 1/3 for Bike and 1/6 for Run2). Statistical analyses were carried out using GraphPad Prism v.7.0 (GraphPad Software, San Diego, CA, USA) and IBM SPSS v.23.0 (SPSS, Chicago, IL, USA).

## 3. Results

### 3.1. Sex and Discipline/Transition Effects on Relative Time

**Figure 1:**
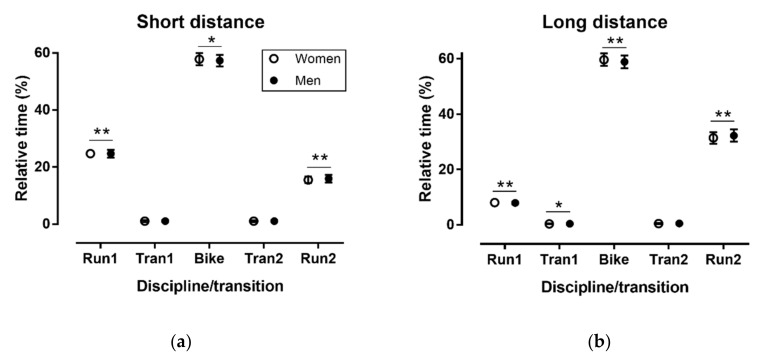
Relative time (%) in disciplines/transitions by sex in short (**a**) and long distance (**b**). Tran = transition; * *p* < 0.01, ** *p* < 0.001.

In the short distance race, a large main effect of discipline/transition on relative time was observed (*p* < 0.001, η^2^_p_ = 0.993) with most time spent in Bike (57.4 ± 2.1%) and the less in the Tran1 (1.0 ± 0.4%) and Tran2 (1.0 ± 0.3%) (Figure 1). A trivial discipline/transition × sex interaction on relative time was shown (*p* < 0.001, η^2^_p_ = 0.007), where women spent more time in Bike (57.8 ± 2.1% versus 57.3 ± 2.1%, respectively) and less in Tran2 (1.0 ± 0.3% versus 1.1 ± 0.3%, respectively) and Run2 than men (15.5 ± 1.2% versus 15.9 ± 1.4%, respectively).

In the long distance race, a large main effect of discipline/transition on relative time was found (*p* < 0.001, η^2^_p_ = 1.000) with most time spent in Bike (59.0 ± 2.3%) and the least in Tran1 (0.4 ± 0.1%) and Tran2 (0.5 ± 0.2%). A small discipline/transition × sex interaction on relative time was observed (*p* < 0.001, η^2^_p_ = 0.029), where women spent more time in Run1 (8.1 ± 0.5% versus 7.9 ± 0.5%, respectively) and Bike (59.6 ± 2.3% versus 58.9 ± 2.3%, respectively), and less time in Tran1 (0.39 ± 0.12% versus 0.41 ± 0.15%, respectively) and Run2 (31.4 ± 2.1% versus 32.3 ± 2.2%, respectively).

### 3.2. Performance Level

**Figure 2:**
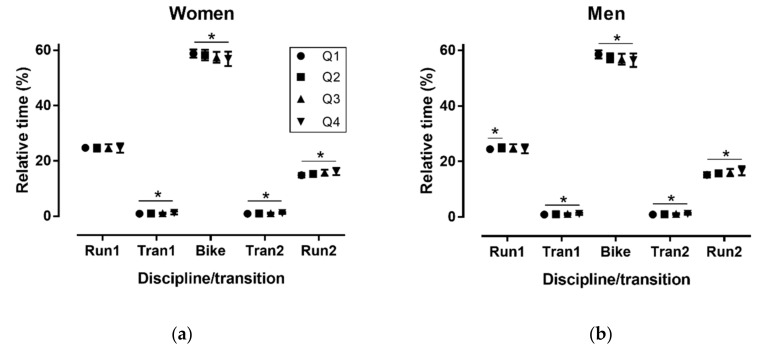
Relative time (%) in disciplines/transitions by performance quartiles in women (**a**) and men (**b**) in the short distance. Q = performance quartile with Q1 the fastest and Q4 the slowest; Tran = transition; * *p* < 0.001.

In the short distance race, in women a medium discipline/transition × quartile interaction on relative time was observed (*p* < 0.001, η^2^_p_ = 0.103), where Q1 was relatively the fastest in Tran1, Tran2 and Run2, and the slowest in Bike (*p* < 0.001), whereas no difference was shown in Run1 (*p* = 0.787) (Figure 2). In men, a medium discipline/transition × quartile interaction on relative time was found (*p* < 0.001, η^2^_p_ = 0.119), where Q1 was relatively the fastest in Run1, Tran1, Tran2 and Run2, and the slowest in Bike (*p* < 0.001).

**Figure 3:**
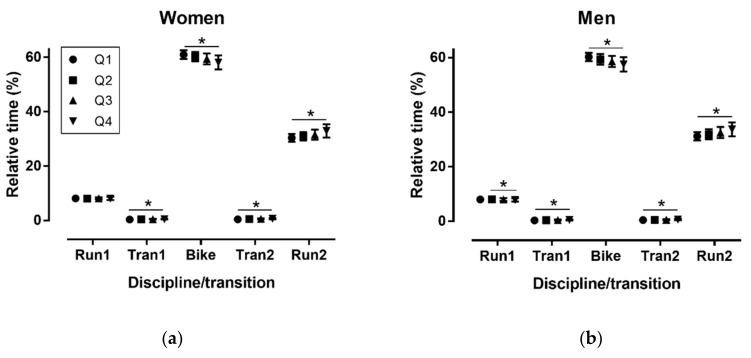
Relative time (%) in disciplines/transitions by performance quartiles in women (**a**) and men (**b**) in the long distance. Q = performance quartile with Q1 the fastest and Q4 the slowest; Tran = transition; * *p* < 0.001.

In the long distance race, in women a large discipline/transition × quartile interaction on relative time was observed (*p* < 0.001, η^2^_p_ = 0.208), where Q1 was relatively the fastest in Tran1, Tran2 and Run2, and the slowest in Bike (*p* < 0.001), whereas no difference was shown in Run1 (*p* = 0.676) (Figure 3). In men, a large discipline/transition × quartile interaction on relative time was found (*p* < 0.001, η^2^_p_ = 0.180), where Q1 was relatively the fastest in Tran1, Tran2 and Run2, and the slowest in Bike (*p* < 0.001).

## 4. Discussion

The main findings of the present study were that (i) women were relatively faster in Run2 and slower in Bike than men in the short and long version of Powerman World Championship; (ii) in short distance, women were faster in Tran2 than men; (iii) in long distance, women were faster in Tran1 and slower in Run1; and (iv) the fastest women and men were the relatively fastest in Run2, Tran1 and Tran2, and the slowest in Bike in both distances.

In both distances, women were relatively slow in Bike and fast in Run2 (i.e., last discipline), which indicated a different distribution of energy expenditure from men across the race. The relatively slow Run2 in men suggested that men decreased performance in the last discipline more than women. That is, it might be supported that women adopted a relatively more even pacing than men across the race, a trend which was in agreement with observations in other endurance sports [7,17,18,19,20,21]. For instance, men slowed more than women in the 5 km Virginia State Championship high school cross-country running race [17] and in the ‘Bolder Boulder’ 10 km road running race [18]. Women also slowed less in Olympic and World Championship marathons [19]. In a 100-km running race in the World Masters Championships, women showed lower relative starting running speeds and higher finishing running speeds than men [20]. In another multi-sport, women maintained and men decreased performance in the last day of ‘Ultraman Hawaii’, a 3-day race [7]. An explanation of the sex difference in pacing might be that this reflects a sex difference in some aspect of decision making, such as overconfidence, risk perception, or willingness to tolerate discomfort [17].

With regards to performance, fast duathletes were the fastest in Run2 and the slowest in Bike, suggesting that their effort was allocated across the race differently from their slower counterparts. Since fast duathletes were the fastest in Run2, it might be assumed that they decreased performance in the last discipline less than their slower peers. This observation might be interpreted as a more even pacing in fast duathletes, which was in line with previous studies in other endurance sports [13,20,21]. For instance, it has been shown that the smaller the deviations of the mean speed during the marathon, the better the performance [21]. In the ‘New York City Marathon’, a lower variability of running speed through the race was found in the top runners for speed during 5 km splits, compared with the less successful runners [13]. Additionally, in a 100-km running race in the World Masters Championships, the ‘top’ competitors displayed lower relative speeds than the ‘bottom’ competitors in the early stages, but higher relative speeds in the later stages of the race [20].

Considering a previous study in triathlon showing that the fastest men were relatively faster in running than in cycling [7], this observation was confirmed in both women and men duathletes in the present study. The relatively faster performance of the fastest performance group in running after cycling highlighted the importance of the final running discipline in duathlon. The role of the final running discipline in duathlon was in agreement with previous observations in Olympic distance triathlon, where performance in running correlated very largely (women) to almost perfectly (men) with overall race time [22].

Duathlon is a self-paced exercise, where the exercise work rate is regulated by the brain based on the integration of numerous signals from various physiological systems [23]. The findings of the present study on the effect of performance on pacing confirmed the observation that during prolonged events (>2 min), performance times may be improved if athletes distribute their pace more evenly [11]. It has been suggested that the most important factor allowing the establishment of a pacing strategy is knowledge of the endpoint of a particular event [24]; thus, the best duathletes may be more experienced and be more aware of this endpoint than their slower counterparts.

A limitation of the findings was that they concerned two races of specific distance; thus, they should be generalized with caution to races of different distance. A strength of the study was its novelty, as it was the first one to examine the effects of sex and performance on pacing in duathlon. Despite the differences of the two versions of ‘Powerman Zofingen’ in terms of total and each discipline’s distance, both presented similar patterns in pacing by sex and performance. These findings have practical applications in this multi-sport as coaches and fitness trainers usually work with small groups of athletes varying for sex and performance. Consequently, these practitioners should develop sex- and performance-tailored training programs and pacing strategies for the race according to the results of the present study.

## 5. Conclusions

In summary, a similar trend of variation of pacing by performance level was observed in both sexes and distances, in which the fastest duathletes performed relatively the fastest Run2 and both transitions, and the slowest Bike. Based on these findings, the role of performance level for adopting a pacing strategy was highlighted, and this role was influenced neither by the total length of duathlon nor by the separate length of each distance.
